# Retraction: Mitochondrial dysfunction and cell death induced by Toona sinensis leaf extracts through MEK/ERK signaling in glioblastoma cells

**DOI:** 10.1371/journal.pone.0349173

**Published:** 2026-05-12

**Authors:** The PLOS One Editors

After this article [1] was published, concerns were raised about the following:

- In Fig 2C, the bottom A172 TSL 40 and 80 µg/ml panels partially overlap.
- No animal ethics approval number is reported, and the article [1] does not provide information on humane endpoints or details regarding euthanasia procedures for the mice.
- Contrary to the Data Availability statement in [1], the individual-level underlying data supporting the published results are not provided with the article.

In response to the concerns about Fig 2C, the corresponding author stated that Fig 2C had been incorrectly assembled and provided the original uncropped images in triplicate along with a replacement figure. They also indicated that the bottom set of six panels in Fig 2C was mislabeled and represents results obtained from the U251 cell line instead.

The corresponding author provided the animal ethics approval document 2024-SH-017 for editorial review. They stated that the mice were monitored at least twice weekly, and that the mice were euthanized with carbon dioxide gas when tumor volume reached approximately 2,000 mm³, when animals exhibited moribund status, or when signs of poor health were observed.

PLOS assessed the individual-level data provided for editorial review which raised additional concerns, including discrepancies between the number of repeats reported in the article and the number of individual data points presented in the underlying data set, discrepancies between the underlying data and values presented in the published article, and irregularities in the tumor volume reporting. The authors’ responses to these issues did not resolve the journal’s concerns.

In light of the unresolved concerns with the underlying data, which call into question the reliability and integrity of the reported results, the *PLOS One* Editors retract this article.

YFS and CYT agreed with the retraction. THT, KLK, CHW, HYS, WCC, FLH, ASL, ALK, JKL, and CLL either did not respond directly or could not be reached.
